# Tanshinone IIA inhibits oral squamous cell carcinoma via reducing Akt-c-Myc signaling-mediated aerobic glycolysis

**DOI:** 10.1038/s41419-020-2579-9

**Published:** 2020-05-18

**Authors:** Ming Li, Feng Gao, Qing Zhao, Huilan Zuo, Wenbin Liu, Wei Li

**Affiliations:** 1 Cell Transplantation and Gene Therapy Institute, The Third Xiangya Hospital, Central South University, 410013 Changsha, Hunan P.R. China; 2 Changsha Stomatological Hospital, 410004 Changsha, Hunan P.R. China; 3 School of Stomatology, Hunan University of Chinese Medicine, 410208 Changsha, Hunan P.R. China; 4 Xiangya Stomatological Hospital & School of Stomatology, Central South University, 410000 Changsha, Hunan P.R. China; 5 Department of Ultrasonography, The Third Xiangya Hospital of Central South University, 410013 Changsha, Hunan P.R. China; 6 Department of Pathology, Hunan Cancer Hospital, 410013 Changsha, Hunan P.R. China; 7 Department of Radiology, The Third Xiangya Hospital of Central South University, 410013 Changsha, Hunan P.R. China

**Keywords:** Enzyme mechanisms, Oral cancer

## Abstract

Aerobic glycolysis is one of the hallmarks of human cancer cells. Overexpression of hexokinase 2 (HK2) plays a crucial role in the maintaining of unlimited tumor cell growth. In the present study, we found that the oral squamous cell carcinoma (OSCC) cells exhibited an aerobic glycolysis phenotype. Moreover, HK2 is highly expressed in OSCC patient derived-tissues and cell lines. Depletion of HK2 inhibited OSCC cell growth in vitro and in vivo. With a natural product screening, we identified Tanshinone IIA (Tan IIA) as a potential anti-tumor compound for OSCC through suppressing HK2-mediated glycolysis. Tan IIA decreased glucose consumption, lactate production, and promoted intrinsic apoptosis in OSCC cells. The mechanism study revealed that Tan IIA inhibited the Akt-c-Myc signaling and promoted E3 ligase FBW7-mediated c-Myc ubiquitination and degradation, which eventually reduced HK2 expression at the transcriptional level. In summary, these results indicate that targeting HK2-mediated aerobic glycolysis is a promising anti-tumor strategy for OSCC treatment.

## Introduction

Oral squamous cell carcinoma (OSCC) is one of the most common types of human oral malignancies, and the incidence and mortality have been increased over the past decades^1,2^. Accumulating evidence has revealed that the genetic susceptibility, life-long history of cigarette smoking, alcohol use, and betel nut chewing are closely related to the increased OSCC risk^3–5^. Even surgery treatment represents the mainstay for early cases, OSCC is often diagnosed at an advanced stage, and metastasis remains the major reason to cause therapy failure, and the 5-year overall survival (OS) of OSCC patients was <50%^6–8^. Currently, no targeted therapy is available for OSCC treatment. Thus, further elucidate the underlying mechanism of OSCC tumorigenesis, and the discovery of novel anti-tumor targets and agents, are still urgent demands for OSCC prevention and treatment.

Deregulation of glycolysis is frequently observed in human cancer cells. The tumor cells preferentially take glycolysis, but not Krebs cycle, as their energy source even in the presence of oxygen^9,10^. Glycolysis is catalyzed by a series of enzymes, including Hexokinase (HK), a rate-limiting enzyme that is required for the first irreversible step to phosphorylate glucose to glucose-6-phosphate. Currently, four HK isozymes have been identified, and only HK2 was overexpressed in multiple human cancer types^11,12^. The high protein level of HK2 increases glucose/nutrient uptake and promotes macromolecular biosynthesis, which is required for sustaining of unlimited tumor cell growth^13,14^. Beyond the critical role in glucose metabolism, HK2 can form a complex with voltage-dependent anion channel (VDAC) on the outer mitochondrial membrane to reduce the release of cytochrome c, which eventually confers therapeutic resistance and promotes cancer cells survival^14–16^. Thus, HK2 is a promising anti-tumor target for cancer treatment.

In this study, with a natural product screening, we identified Tanshinone IIA (Tan IIA), a major component isolated from Danshen (*Salvia miltiorrhiza Bunge*), as a potential anti-cancer compound for OSCC treatment. We examined the inhibitory effect of Tan IIA on OSCC cells both in vitro and in vivo and investigated the underlying mechanism of this anti-tumor activity.

## Materials and methods

### Cell culture and antibodies

The SDS, DMSO, NaCl, and Tris base, which was used for buffer preparation, were purchased from Sigma-Aldrich (St. Louis, MO). The proteasome inhibitor, MG132, and cycloheximide (CHX) were obtained from Thermo Fisher Scientific (Waltham, MA). Fetal Bovine Serum (FBS) and cell culture media, such as DMEM and RPMI-1640 medium, were products of Invitrogen (Grand Island, NY). Human oral squamous cell carcinoma (OSCC) cells, including SCC9 SCC15, SCC25, and CAL27, were purchased from American Type Culture Collection (ATCC, Manassas, VA). Immortalized oral epithelial cell hTERT-OME was purchased from Applied Biological Materials (ABM) Inc. (Richmond, BC, Canada). All cells were maintained at the 37 °C humidified incubator with 5% CO_2_ according to ATCC protocols and subjected to mycoplasma test every two months. Antibodies against HK2 (#2867), c-Myc (18583), HK1 (#2024), p-Akt (#4060), Akt1 (#2938), p-GSK3β (#5558), GSK3β (#12456), Bax (#14796), VDAC1 (#4866), α-Tubulin (#2144), Cytochrome c (#4280), cleaved-caspase 3 (#9664,), β-actin (#3700), Ubiquitin (#3936, #43124), cleaved-PARP (#5625), anti-rabbit IgG HRP (#7074), and anti-mouse IgG HRP (#7076) were purchased from Cell Signaling Technology, Inc. (Beverly, MA). Antibodies against Ki67 (ab16667) was from Abcam (Cambridge, UK). FBW7 (40–1500) antibody was purchased from Thermo Fisher Scientific (Waltham, MA). Antibody conjugates were visualized by chemiluminescence (#34076, Thermo Fisher Scientific).

### Natural product screening

The Natural Product Library (Cat. No. L1400-01/02) is a product of Selleck Chemicals (Houston, TX), and the 88 compounds of interest (Supplementary Table 1) used for screening were selected from this Natural Product Library. CAL27 cells were plated in a 48-well plate and treated with a single dose of 2 μM natural compounds or DMSO (control) for 12 h. The supernatant of the cell culture medium was subjected to glucose and lactate analysis at the Laboratory of Xiangya Hospital (Changsha, China). The compound-treated CAL27 cells were concentrated with BCA protein assay and used as a loading control for glucose consumption and lactate production rate normalization. All screened compounds are listed in Table S1. There are two reasons for us to select Tan IIA as a candidate compound for the present study. Firstly, based on the natural product screening, only Tan IIA decreased both glucose consumption and lactate production over 25% at the concentration of 2 μM, indicating that Tan IIA possesses the most substantial inhibitory effect on glycolysis. Secondly, Tan IIA is a major component of traditional Chinese herb, Danshen, and currently used for clinical treatment of arecoline- and areca nut extract-induced oral submucous fibrosis (OSF), which is an oral precancerous lesion. Thus, it’s worthy of studying the underlying anti-tumor mechanisms of Tan IIA in OSCC.

### MTS assays

MTS assay was performed as described previously^17^. OSCC cells were seeded into a 96-well plate at a concentration of 3000 cells per well. After overnight incubation, cells were treated with Tanshinone IIA for various time points. Cell viability was examined with the Cell Proliferation Assay kit (MTS) purchased from Promega (#G3580, Madison, WI) following the manufacturer’s instructions.

### Anchorage-independent cell growth

The anchorage-independent cell growth assay was performed as described previously^18^. Briefly, the Eagle’s basal medium containing 10% FBS/0.5% agar was prepared in the six-well plates as a culture layer. OSCC cells were counted and diluted with the 10%FBS/0.3% agar containing Eagle’s basal medium to a final concentration of 8000 cells/ml and overplayed into the six-well plates with bottom culture layer. The colony number was counted after 2 weeks culture at 37 °C in a 5% CO_2_ incubator.

### Clinical tissue sample collections

A total of 20 cases of OSCC tissues and matched adjacent non-tumor tissues were collected from 20 patients with written informed consent by the Department of pathology, Hunan Cancer Hospital of Central South University, Changsha, Hunan, China. All the patients received no treatment before surgery. The samples were frozen in liquid nitrogen, and protein was prepared for western blotting analysis.

### Western blotting

The whole-cell extract (WCE) was prepared using the commercial RIPA buffer (#PI89901) from Thermo Fisher Scientific. WCE was concentrated by BCA protein assay (#23228, Thermo Fisher Scientific) and subjected to IB analysis as described previously^19^. Briefly, a total of 30 μg of WCE was boiled with loading buffer for 5 min and subjected to SDS-PAGE electrophoresis and electrotransfer to the PVDF membrane. The membrane was blocked with 5% non-fat milk at room temperature for 1 h, followed by incubation with primary antibody at 4 °C overnight and second antibody incubation at room temperature for 30 min. The target protein was visualized by chemiluminescence.

### HK2 knock out stable cell lines

Two different single-guide RNAs (sgRNAs) were used to generate HK2, Akt1, and c-Myc stable knockout cells (Supplementary Table 2). The sgHK2 plasmid was transfected to the OSCC cells and selected by puromycin (1 μg/mL) for 3 weeks. A single colony was used for further study.

### Glycolysis analysis

The glycolysis analysis was performed as described previously^20^. Briefly, OSCC cells were seeded in 48-well plates and treated with a DMSO (control) or Tanshinone IIA for 12 h. Glucose consumption and lactate production were analyzed at the Laboratory of Hunan Cancer Hospital of Central South University (Changsha, China). The relative glucose consumption and lactate production rate were normalized by protein concentration.

### Ubiquitination analysis

The Ubiquitination analysis was performed as described previously^21^. For IP-mediated endogenous ubiquitination analysis, the cells were lysed with modified RIPA buffer (20 mM NAP, pH7.4, 150 mM NaCl, 1% Triton, 0.5% Sodium-deoxycholate, and 1% SDS) supplemented with 10 mM *N*-ethylmaleimide (NEM) and protease inhibitors. The lysates were sonicated for 30 s, boiled at 95 °C for 15 min, and diluted with 0.1% SDS containing RIPA buffer, then centrifuged at 16,000 × *g* for 15 min at 4 °C. The supernatant was subjected to IP assay, followed by IB analysis of target protein ubiquitination. For Nickel pull-down assay, cells were lysed with Ni-NTA lysis buffer (6 M guanidine-HCl, 0.1 M Na2HPO4/NaH2PO4, 0.01 M Tris/HCl, pH 8.0, 5 mM imidazole, and 10-mMβ-mercaptoethanol) supplemented with protease inhibitors and 10 mM *N*-ethylmaleimide (NEM). The lysates were sonicated for 30 s, followed by incubation with 50 ml Ni-NTA-agarose (QIAGEN Inc, Valencia, CA) for 4 h at room temperature. After the last wash, the Ni-NTA beads were boiled with loading buffer containing 200 mM imidazole. Ubiquitination was determined by IB analysis.

### In vivo tumor growth

All animal experiments were approved by the Institutional Animal Care and Use Committee (IACUC) of Central South University (Changsha, China). The OSCC xenograft models were constructed by s.c.injection of CAL27 (2 × 10^6^) or SCC15 (5 × 10^6^) cells into the right flank of 6-week-old athymic nude mice (*n* = 5). Tumor volume and mouse body weight were recorded every 2 days. The tumor-bearing mice were initiated with compound treatment when the tumor volume reached ~100 mm^3^. The mice were divided into two groups randomly. The control group was administrated the vehicle control, whereas the compound-treated group was administrated Tanshinone IIA (10 mg/kg every two days) by i.p. injection. Tumor volume was determined according to the following formula: length × width × width/2. At the endpoint, tumor mass was fixed and subjected to immunohistochemical (IHC) staining.

### Immunohistochemical staining

Mice xenograft tumors were fixed and subjected to IHC analysis as described previously^22^. Briefly, the tissue slides were deparaffinized and rehydrated by subsequently incubation with xylene and ethanol to complete the removal of paraffin. Antigen retrieval was performed by submerging the tissue slides into sodium citrate buffer (10 mM, pH 6.0) and boiled for 10 min. After a wash with ddH_2_O for three times, the slides were incubated with 3% H_2_O_2_ in methanol for 10 min to deactivate the endogenous horseradish peroxidase, followed by washing with PBS for three times. The slides were blocked with 50% goat serum albumin in PBS at room temperature for 1 h and hybridized with the primary antibody in a humidified chamber overnight at 4 °C. Tissue slides were incubated with secondary antibody at room temperature for 45 min and visualized by DAB substrate. Hematoxylin was used for counterstaining.

### Blood analysis

Mouse blood was collected by cardiac puncture into the EDTA-coated tubes. The red blood cells (RBC), white blood cells (WBC), hemoglobin (Hb), alanine aminotransferase (ALT), aspartate aminotransferase (AST), and blood urea nitrogen (BUN) were analyzed at the Laboratory of the Third Xiangya Hospital of Central South University (Changsha, China).

### Statistical analysis

Statistical analysis was performed using GraphPad Prism 5 (GraphPad 5.0, San Diego, CA, USA). The quantitative data are expressed as mean ± sd. The difference was evaluated using the Student’s *t*-test or ANOVA. A probability value of *p* < 0.05 was used as the criterion for statistical significance.

## Results

### Highly expressed HK2 is required for maintaining of tumorigenic properties of OSCC cells

**Fig. 1:**
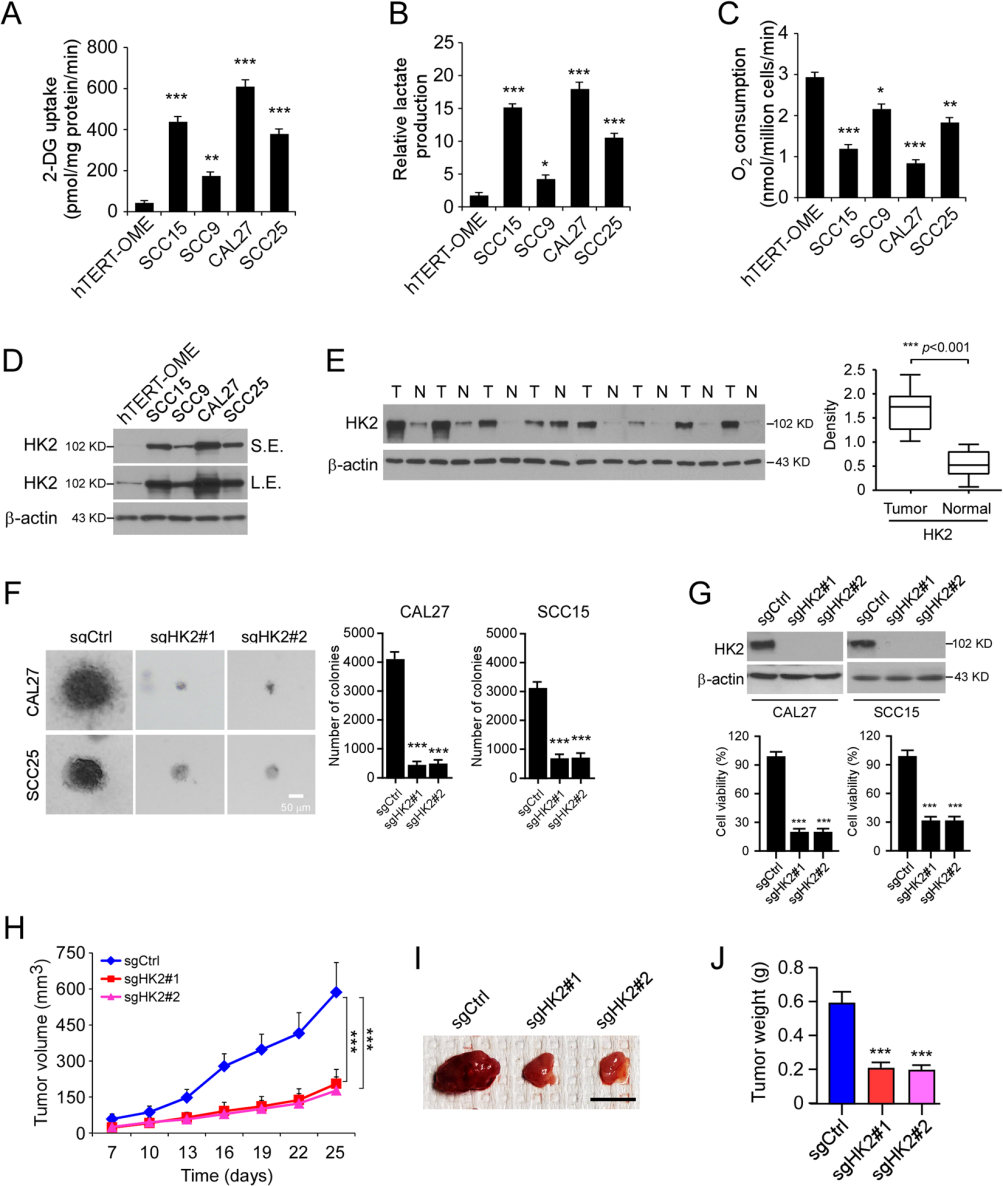
HK2 is highly expressed in oral squamous cell carcinoma (OSCC) cells. **a**–**c** Normalized 2-DG uptake (**a**), lactate production (**b**), and O_2_ consumption (**c**) in a panel of OSCC cells (SCC15, SCC9, CAL27, and SCC25) and normal oral epithelial cells under normoxic conditions. **d** Immunoblotting (IB) analysis of HK2 expression in OSCC and hTERT-OME cells. **e** The representative IB results of HK2 expression in 30 cases of matched OSCC patient tissues and adjacent tissues. T tumor, N adjacent non-tumor tissue. **f** Colony formation of CAL27 and SCC15 cells expressing sgCtrl or sgHK2. Scale bar, 50 µm. **g** Cell viability of CAL27 and SCC15 cells expressing sgCtrl or sgHK2. Top, IB analysis of HK2 expression. Bottom, MTS analysis of cell viability. **h**–**j** In vivo tumor growth of CAL27 cells expressing of sgCtrl or sgHK2. **h** tumor volume. **i** The image of the tumor mass. Scale bar, 1 cm. **j** The weight of the tumor mass. *p < 0.05, **p < 0.01, ***p < 0.001.

To investigate the glucose metabolic characteristics of human OSCC cells, we examined the glycolysis efficacy of four OSCC cells and the immortalized oral epithelial cells in normoxic culture conditions. Our data showed that the CAL27 cells exhibited the highest glycolysis efficacy as the 2-DG uptake (Fig. 1a) and lactate production (Fig. 1b) were significantly increased when compared to that of the immortalized oral epithelial cell hTERT-OME. Also, the O_2_ consumption ratio was significantly decreased in CAL27 and other OSCC cells (Fig. 1c), indicating that the oxidative phosphorylation was inhibited in OSCC cells. These results suggest that glycolysis was used as a major glucose metabolism pathway in the tested OSCC cell lines. HK2 catalyzes the first rate-limiting step of aerobic glycolysis. We speculated that HK2 might play a role in the upregulated glycolysis in OSCC cells. Thus, we first determined the protein level of HK2 in OSCC cells and immortalized oral epithelial cells. As shown in Fig. 1d, HK2 is overexpressed in human OSCC cell lines, including CAL27, SCC25, SCC15, and SCC9, and CAL27 cells exhibited the most robust HK2 expression. We further examined the expression of HK2 in 20 cases of paired OSCC and adjacent tissues. Our data showed that the protein level of HK2 is significantly upregulated in OSCC tissues (Fig. 1e). These results indicate that the cells in which expression of higher protein levels of HK2 possessed more substantial glycolysis potential. We next generated HK2 knockout stable cell lines using HK2 highly expressed CAL27 and SCC15 cells. The results revealed that the depletion of HK2 inhibited the colony formation of OSCC cells in soft agar significantly (Fig. 1f). Furthermore, knockout of HK2 caused the decrease of cell viability in CAL27, and SCC15 cells were confirmed by the MTS assay (Fig. 1g). Importantly, knockout of HK2 inhibited glycose consumption and lactate production in CAL27 and SCC15 cells (Supplementary Fig. 1A, B). In contrast, overexpression of HK2 in immortalized epithelial cell hTERT-OME, increased glucose consumption and lactate production (Supplementary Fig. 1C, D). The xenograft mouse model showed that the depletion of HK2 caused a significant delay of in vivo tumor development of CAL27-derived xenograft tumors (Fig. 1h–j). These results indicate that HK2 is overexpressed in OSCC tissues and cell lines, knockout of HK2 reduces the tumorigenic properties of OSCC cells.

### Tan IIA inhibits glycolysis of OSCC cells through the downregulation of HK2

**Fig. 2:**
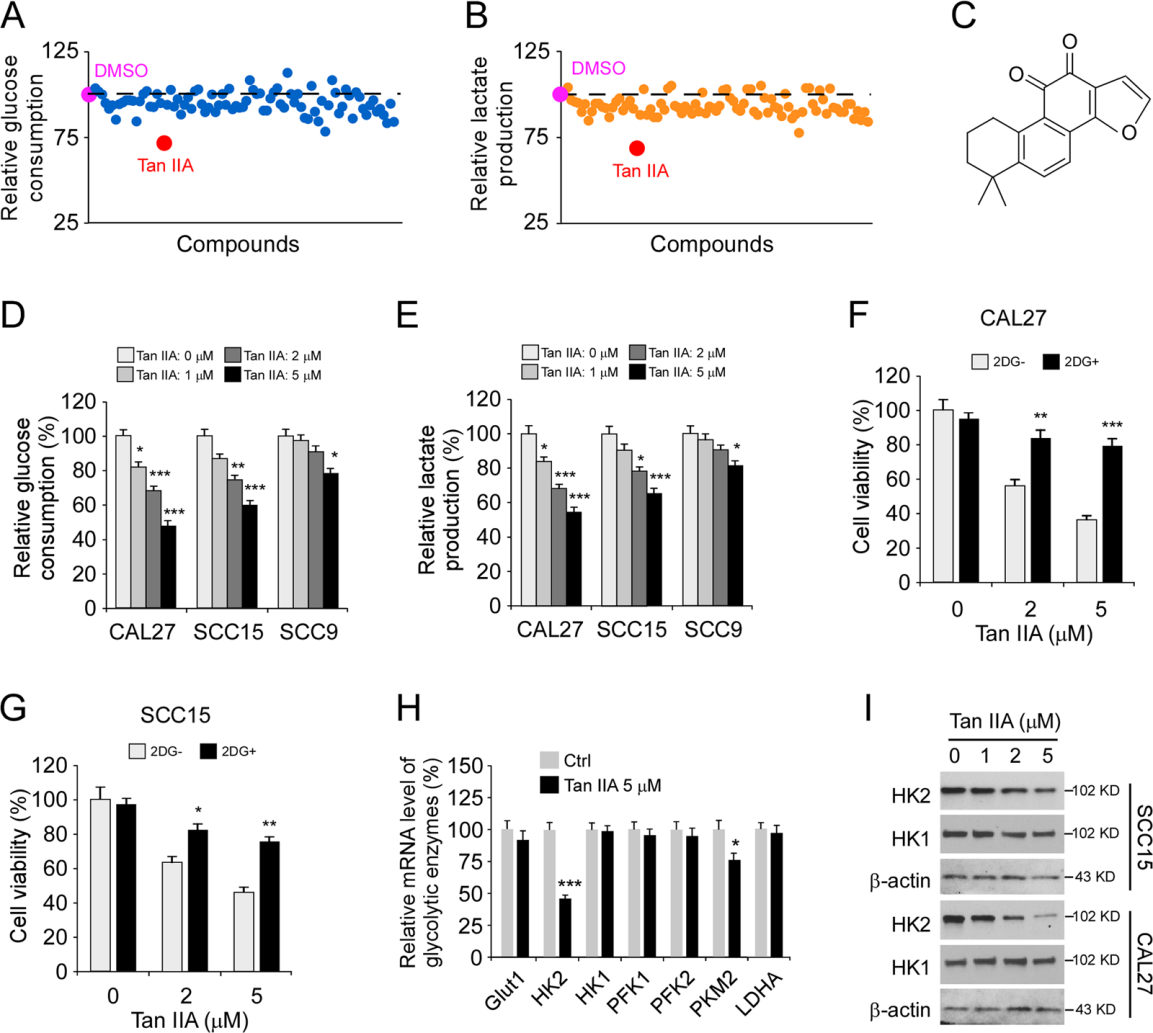
Tanshinone IIA (Tan IIA) inhibits glycolysis in OSCC cells. **a**, **b** The inhibitory efficacy of screened compounds on glucose consumption (**a**) and lactate production (**b**) of CAL27 cells. **c** The chemical structure of Tan IIA. **d**, **e** The inhibitory efficacy of Tan IIA on glucose consumption (**d**) and lactate production (**e**) of OSCC cells. **f**, **g** MTS analysis the cell viability of CAL27 (**f**) and SCC15 (**g**) cells treated with 2-DG, Tan IIA, or combination treatment. **h** qRT-PCR analysis of the mRNA level of glycolytic enzymes with Tan IIA treatment. **i** IB analysis of HK2 and HK1 expression in Tan IIA-treated CAL27 and SCC15 cells. *p < 0.05, **p < 0.01, ***p < 0.001.

To discover the natural compounds that can suppress glycolysis and HK2 in OSCC cells, we performed a natural product screening using CAL27 cells. The results showed that only Tan IIA decreased glucose consumption and lactate production (Fig. 2a–c) over 25% when compared to that of DMSO treated cells. We then focused on Tan IIA for further study. To confirm the inhibitory effect of Tan IIA on glycolysis, we treated three OSCC cells, which expresses high (CAL27), moderate (SCC15), or low (SCC9) level of HK2 with various dose of Tan IIA. The results showed that Tan IIA reduced glucose consumption (Fig. 2d) and lactate production (Fig. 2e) dose-dependently. Moreover, Tan IIA exhibited the strongest inhibitory efficacy against CAL27 cells than that of SCC15 and SCC9 cells. The SCC9 cells are relatively resistant to Tan IIA treatment, and only 5 µM Tan IIA reduced glycolysis significantly (Fig. 2d, e). These results suggest that the OSCC cells which addicted to glycolysis are more sensitive to Tan IIA treatment. Importantly, treatment with Tan IIA significantly increased O_2_ consumption in CAL27 and SCC15 cells dose-dependently (Supplementary Fig. 2), indicating that Tan IIA restored oxidative phosphorylation in OSCC cells. Based on these results, we speculate that suppression of glycolysis may compromise the inhibitory effect of Tan IIA on aerobic glycolytic OSCC cells. We then treated CAL27 and SCC15 cells with glycolysis inhibitor 2-DG or DMSO control. The results revealed that Tan IIA decreased the cell viability of both 2-DG and DMSO treated OSCC cells (Fig. 2f, g). Moreover, treated with 2-DG attenuated the inhibitory effect of Tan IIA on CAL27 and SCC15 cells. Importantly, 2-DG rescued the inhibitory effect much stronger in CAL27 cells than that of SCC15 (Fig. 2f, g). These results further confirmed that the OSCC cells, which exhibit a high glycolysis rate, are more sensitive to Tan IIA treatment. We next examined the mRNA level of multiple glycolytic enzymes after Tan IIA treatment (Fig. 2h). Our data showed that the transcription of HK2 was reduced the most among the tested enzymes. Furthermore, Tan IIA also decreased the mRNA level of PKM2 rather than HK1, Glut1, PFK1/2, and LDHA (Fig. 2h). The IB results further confirmed that Tan IIA inhibited the expression of HK2, but not HK1, in both CAL27 and SCC15 cells (Fig. 2i). Our data indicate that Tan IIA is a candidate compound for glycolysis inhibition in OSCC cells by reducing of HK2 expression.

### Tan IIA activates intrinsic apoptosis in OSCC cells

**Fig. 3:**
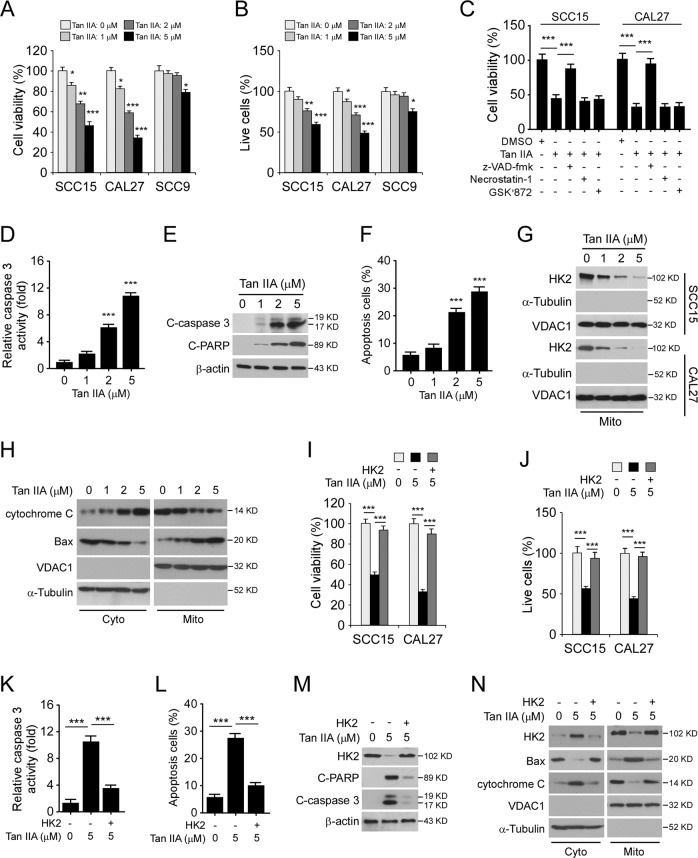
Tan IIA decreases the HK2 protein level and induces intrinsic apoptosis. **a** MTS analysis of the cell viability of OSCC cells treated with Tan IIA. **b** Trypan blue exclusion assay analysis of the population of live cells in Tan IIA-treated OSCC cells. **c** MTS analysis the cell viability of OSCC cells treated with various inhibitors and Tan IIA. **d,** **e** Caspase 3 activity (**d**), and the protein level of cleaved caspase 3 and -PARP (**e**) in Tan IIA-treated CAL27 cells. **f** Flow cytometry analysis of the population of apoptotic cells in Tan IIA-treated CAL27 cells. **g** IB analysis of HK2 expression in the mitochondrial fraction of Tan IIA-treated CAL27 cells. **h** CAL27 cells were treated with Tan IIA, subcellular fractions were isolated and subjected to IB analysis. **i** MTS assay analysis of cell viability of OSCC cells transfected with HK2 and treated with Tan IIA. **j** Trypan blue exclusion assay analysis of the population of live cells in HK2 transfected and Tan IIA-treated OSCC cells. **k** Caspase 3 activity in HK2 transfected and Tan IIA-treated CAL27 cells. **l** Flow cytometry analysis of the population of apoptotic cells in HK2 transfected and Tan IIA-treated CAL27 cells. **m** IB analysis of cleaved caspase 3 and -PARP in HK2 transfected and Tan IIA-treated CAL27 cells. **n** CAL27 cells were transfected with HK2 and treated with Tan IIA for 24 h. Subcellular fractions were isolated and subjected to IB analysis. *p < 0.05, **p < 0.01, ***p < 0.001.

Because the mitochondrial localization of HK2 is required for cell survival and apoptosis suppression, we next examined the cell viability of Tan IIA-treated OSCC. The result showed that Tan IIA reduced cell viability in CAL27, SCC15, and SCC9 cells (Fig. 3a). Importantly, the population of live cells was reduced time- and dose-dependently (Fig. 3b, Supplementary Fig. 1E, F), indicating that treatment with Tan IIA caused cell death in OSCC cells. We next pre-treated OSCC cells with apoptosis inhibitor, z-VAD-fmk, and necroptosis inhibitor necrostatin-1/GSK′872. The results revealed that z-VAD-fmk, but not necrostatin-1 or GSK′872, rescued Tan IIA-induced downregulation of cell viability (Fig. 3c). Furthermore, the activity of caspase 3 (Fig. 3d), and the protein level of cleaved caspase 3 and -PARP (Fig. 3e), were upregulated dose-dependently. The flow cytometry data showed that the population of apoptotic cells was increased with Tan IIA treatment (Fig. 3f). With the isolation of the subcellular fractions, we further confirmed that Tan IIA inhibited the presence of HK2 on mitochondria (Fig. 3g). Moreover, the release of cytochrome C from mitochondria to the cytoplasm was increased dose-dependently, and the expression of mitochondria-associated Bax increased after Tan IIA treatment (Fig. 3h). This evidence suggests that Tan IIA activated the intrinsic apoptosis signaling. To determine whether Tan IIA-induced apoptosis is HK2 dependent, we ectopically overexpressed HK2 in CAL27 and SCC15 cells. The results revealed that overexpression of HK2 rescued cell viability (Fig. 3i), and reduced the population of death cells (Fig. 3j) with Tan IIA treatment. Likewise, the caspase 3 activity (Fig. 3k) and the cell number of apoptotic cells (Fig. 3l) were decreased in HK2 overexpressed OSCC cells. The IB data further confirmed that overexpression of HK2 inhibited the expression of cleaved caspase 3 and -PARP (Fig. 3m), and suppressed Tan IIA-induced activation of mitochondrial apoptosis (Fig. 3n). Overall, these results indicate that Tan IIA activates intrinsic apoptosis in an HK2-dependent manner in OSCC cells.

### Tan IIA promotes ubiquitination-dependent degradation of c-Myc

**Fig. 4:**
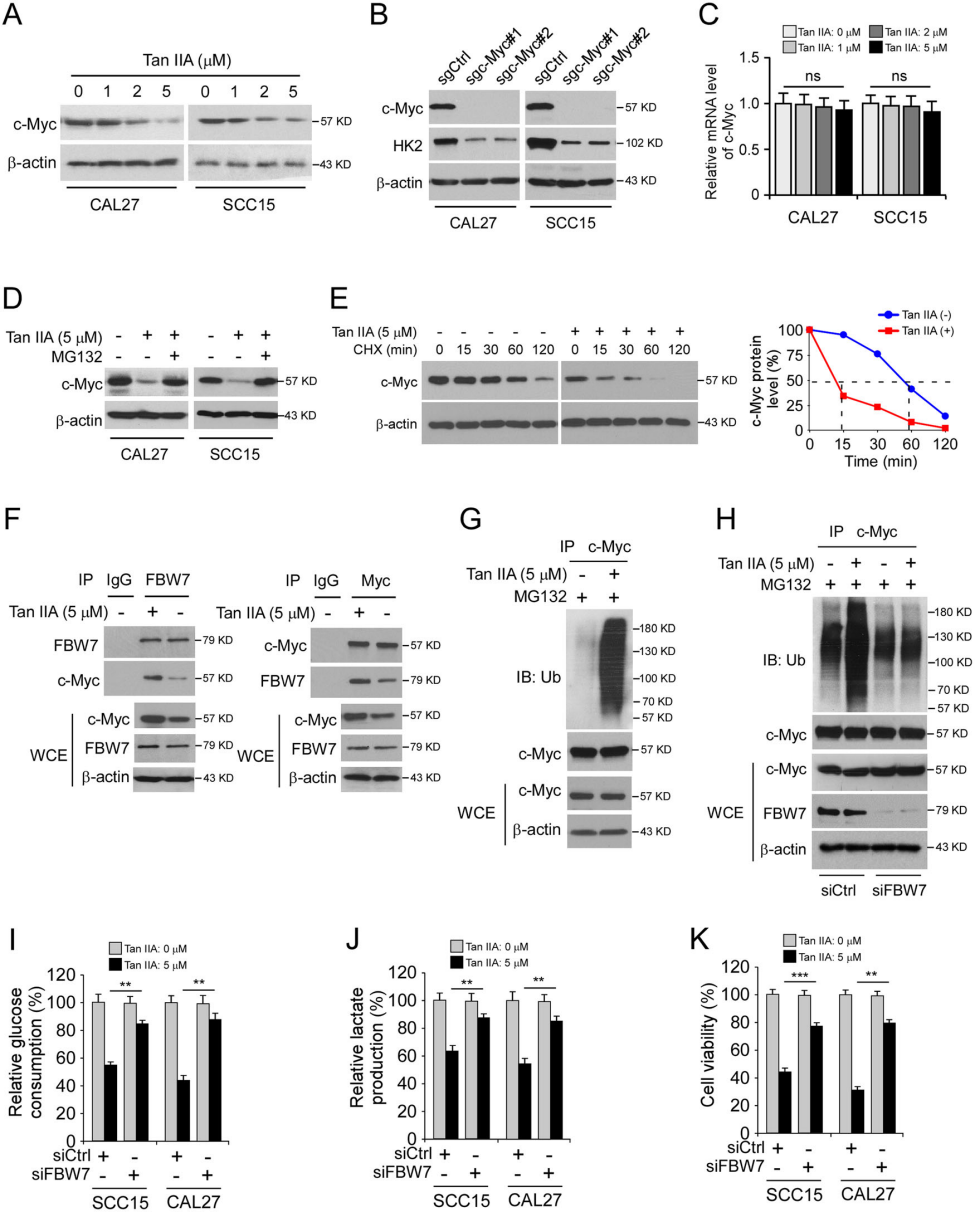
Tan IIA promotes c-Myc ubiquitination and degradation. **a** IB analysis of c-Myc expression in OSCC cells with Tan IIA treatment. **b** IB analysis of c-Myc and HK2 expression in sgCtrl and sgc-Myc expressing OSCC cells. **c** qRT-PCR analysis of c-Myc mRNA level in Tan IIA-treated OSCC cells. ns, not statistically significant. **d** OSCC cells were treated with Tan IIA for 24 h, MG132 was added to the cell culture medium and incubated for another 6 h, whole-cell lysate (WCE) was subjected to IB analysis. **e** OSCC cells were treated with Tan IIA for 24 h, cycloheximide (CHX) was added to the cell culture medium and incubated for various time points as indicated, whole-cell lysate (WCE) was subjected to IB analysis. **f** co-IP analysis of the interaction between c-Myc and FBW7 in Tan IIA-treated CAL27 cells. **g** Ubiquitination analysis of c-Myc ubiquitination in Tan IIA-treated CAL27 cells. **h** CAL27 cells were transfected with siCtrl or siFBW7 shRNA followed by Tan IIA-treated for 24 h, WCE was subjected to ubiquitination analysis. **i**, **j** CAL27 cells were transfected with siCtrl or siFBW7 shRNA followed by Tan IIA-treated for 24 h, the cell culture medium was subjected to glucose consumption (**i**) and lactate production (**j**) analysis. **k** CAL27 cells were transfected with siCtrl or siFBW7 shRNA followed by Tan IIA-treated for 24 h and subjected to MTS assay for cell viability analysis. **p < 0.01, ***p < 0.001.

The oncoprotein c-Myc is one of the most important transcription factors for HK2 expression^23^. Our data showed that Tan IIA decreased the mRNA level of HK2, we thus determined whether Tan IIA inhibits c-Myc in OSCC cells. The results showed that Tan IIA decreased the protein level of c-Myc dose-dependently (Fig. 4a). Moreover, knockout of c-Myc inhibited HK2 expression in OSCC cells (Fig. 4b). The qRT-PCR result indicated that Tan IIA did not suppress the transcription of c-Myc, as the mRNA level of c-Myc in Tan IIA-treated OSCC cells was unaffected (Fig. 4c). In addition, treated with the proteasome inhibitor, MG132, restored c-Myc expression in Tan IIA-treated OSCC cells (Fig. 4d), indicating that Tan IIA promoted c-Myc degradation. Indeed, Tan IIA shortened c-Myc half-life from 1 h to 15 min (Fig. 4e). Because the FBW7 is an E3 ligase which required for c-Myc degradation, we first examined the interaction between c-Myc and FBW7. The co-IP data revealed that Tan IIA promoted the interaction between c-Myc and FBW7 (Fig. 4f). Ubiquitination analysis showed that Tan IIA enhanced c-Myc ubiquitination in CAL27 cells (Figure G). To confirm that Tan IIA-induced c-Myc ubiquitination is dependent on E3 ligase FBW7, we reduced FBW7 expression in CAL27 cells using siRNA. Our data indicate that knockdown of FBW7 attenuated Tan IIA promoted c-Myc ubiquitination (Fig. 4h). We next determined whether knockdown of FBW7 affected glycolysis in OSCC cells. The results showed that the decrease of FBW7 rescued the glycolytic phenotype in OSCC cells, as the glucose consumption (Fig. 4i) and lactate production (Fig. 4j) were restored in FBW7 knockdown OSCC cells. Consistently, knockdown of FBW7 increased cell viability in Tan IIA-treated OSCC cells (Fig. 4k). Overall, these data indicate that Tan IIA promotes FBW7-mediated c-Myc destruction is required for glycolysis suppression in OSCC cells.

### Akt rescued c-Myc expression in Tan IIA-treated OSCC cells

**Fig. 5:**
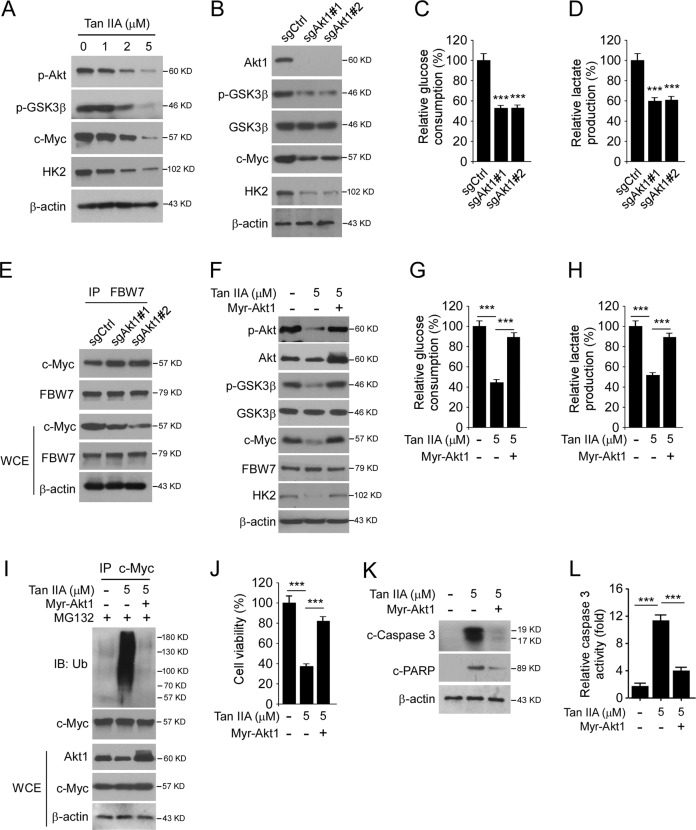
Tan IIA inhibits c-Myc expression in an Akt signaling dependent manner. **a** CAL27 cells were treated with Tan IIA for 24 h, WCE was subjected to IB analysis. **b** The sgCtrl and sgAkt stable expressing CAL27 cells were treated with Tan IIA for 24 h, WCE was subjected to IB analysis. **c**, **d** The sgCtrl, and sgAkt stable expressing CAL27 cells were treated with Tan IIA for 24 h. Cell culture medium was subjected to glucose consumption (**c**) and lactate production (**d**) analysis. **e** The sgCtrl and sgAkt stable expressing CAL27 cells were treated with Tan IIA for 24 h, WCE was subjected to co-IP analysis. **f** CAL27 cells were transfected with Myr-Akt1 and treated with Tan IIA for 24 h, WCE was subjected to IB analysis. **g**, **h** CAL27 cells were transfected with Myr-Akt1 and treated with Tan IIA for 24 h, the cell culture medium was subjected to glucose consumption (**g**) and lactate production (**h**) analysis. **i** CAL27 cells were transfected with Myr-Akt1 and treated with Tan IIA for 24 h, WCE was subjected to ubiquitination analysis. **j** CAL27 cells were transfected with Myr-Akt1 and treated with Tan IIA for 24 h, MTS assay was performed to analyze the cell viability. **k**, **l** CAL27 cells were transfected with Myr-Akt1 and treated with Tan IIA for 24 h, WCE was subjected to IB analysis (**k**) and caspase 3 activity analysis (**l**). ***p < 0.001.

GSK3β-mediated phosphorylation at Thr58 is required for c-Myc ubiquitination and degradation^24^. Our data showed that Tan IIA promoted c-Myc ubiquitination, we speculated that Tan IIA activated GSK3β. The IB data showed that treated with Tan IIA inhibited the phosphorylation of Akt dose-dependently. As a downstream target, the phosphorylation of GSK3β on Ser9 was reduced consistently (Fig. 5a), indicating that the activity of GSK3β was increased. Furthermore, knockout of Akt1 by sgRNA inhibited the phosphorylation of GSK3β on Ser9 and decreased the protein level of both c-Myc and HK2 in OSCC cells (Fig. 5b). Likewise, the glucose consumption (Fig. 5c) and lactate production (Fig. 5d) were attenuated subsequently in Akt1 sgRNA expressing cells. The co-IP data revealed that knockout of Akt1 promoted the interaction between c-Myc and FBW7 (Fig. 5e). We next determined whether the overexpression of Akt1 rescued this phenotype. The IB data showed that ectopic overexpression of constitutively activated Akt1 (Myr-Akt1), restored Akt phosphorylation on S473 even in the presence of Tan IIA (Fig. 5f). Moreover, Myc-Akt1 transfection suppressed the activation of GSK3β, as the phosphorylation of S9 was increased robustly. Also, the protein levels of c-Myc and HK2 were increased consistently (Fig. 5f). The glycolysis analysis results further confirmed that overexpression of Myc-Akt1 enhanced glucose consumption (Fig. 5g) and lactate production (Fig. 5h) in Tan IIA-treated OSCC cells. In addition, Tan IIA-induced polyubiquitination of c-Myc was reduced substantially in Myc-Akt1 transfected cells (Fig. 5i). MTS data revealed that Myc-Akt1 restored cell viability from 40 to 80% when compared to that of the untreated cells (Fig. 5j). Consistently, the protein level of cleaved caspase 3 and -PARP (Fig. 5k), as well as the activity of caspase 3 (Fig. 5l), were reduced significantly in Myc-Akt1 overexpressed OSCC cells. Overall, our data suggesting that Akt rescued c-Myc expression in Tan IIA-treated OSCC cells.

### Tan IIA suppresses the in vivo tumor development of OSCC cells

**Fig. 6:**
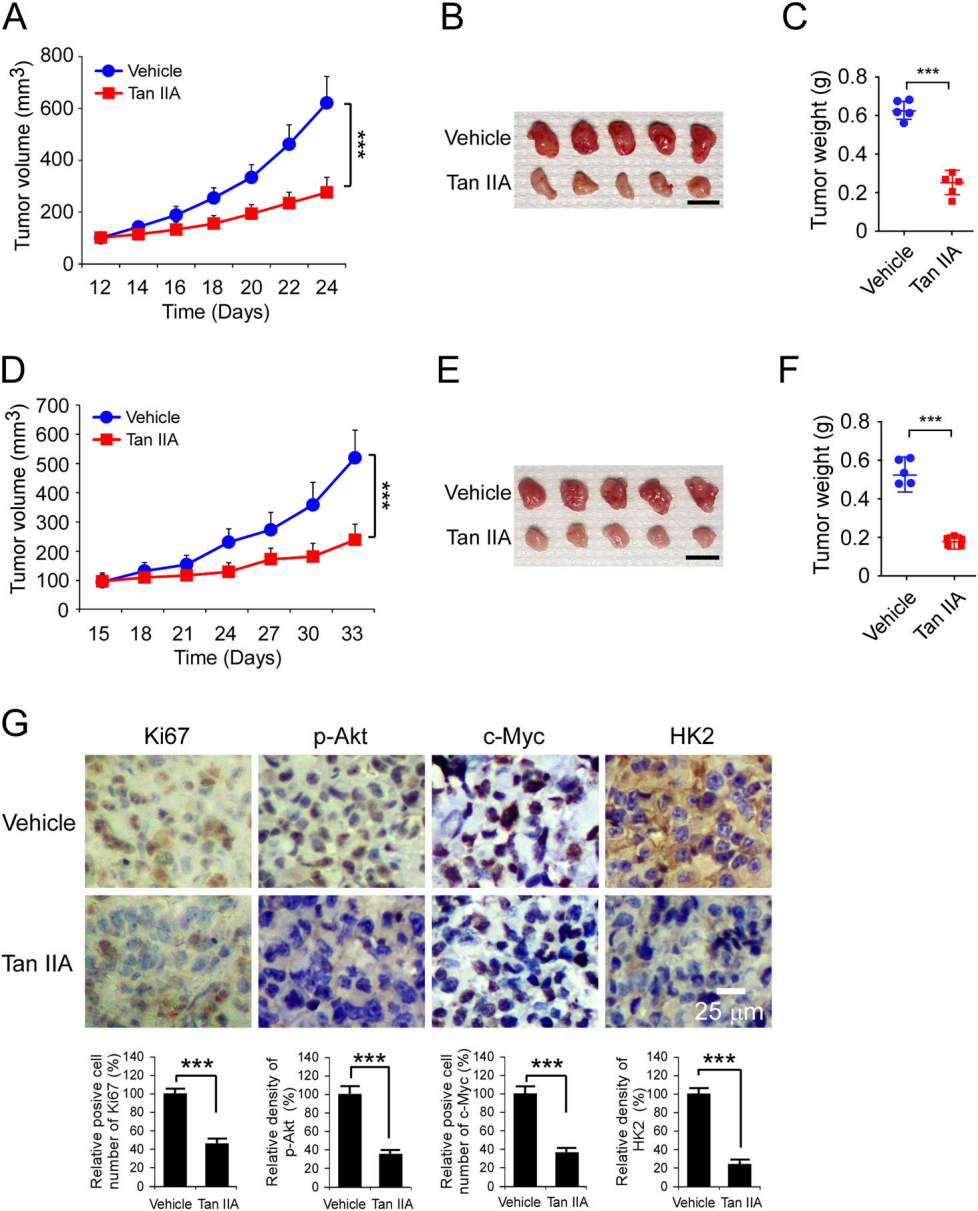
Tan IIA inhibits the in vivo tumor growth of OSCC cells. **a**–**c** The tumor volume (**a**), The image of tumor mass (**b**), and tumor weight (**c**) of CAL27-derived xenograft tumors treated with vehicle or Tan IIA. **d**–**f** The tumor volume (**d**), The image of tumor mass (**e**), and tumor weight (**f**) of SCC15-derived xenograft tumors treated with vehicle or Tan IIA. Scale bar, 1 cm. **g** IHC staining of Ki67, p-Akt, c-Myc, and HK2 in CAL27-derived xenograft tumors with vehicle or Tan IIA treatment. ***p < 0.001. Scale bar, 25 μm.

To further determine the in vivo anti-tumor effect of Tan IIA, we performed a xenograft mouse model using CAL27 and SCC15 cells. The results showed that the tumor volume of the vehicle-treated group of CAL27-derived xenograft tumors was 625 ± 111 mm^3^. In contrast, Tan IIA treatment significantly inhibited the in vivo tumor growth, as the tumor volume was only 281 ± 56 mm^3^ (Fig. 6a). We further examined the tumor weight of both vehicle- and Tan IIA-treated group. The result showed that Tan IIA reduced tumor weight over 50% when compared to that of the vehicle-treated group (Fig. 6b, c). Likewise, we observed a similar inhibitory efficacy of Tan IIA on SCC15-derived xenograft tumors. The results indicated that both tumor volume and tumor weight were significantly decreased in the Tan IIA-treated xenograft tumors (Fig. 6d–f). IHC staining revealed that administration with Tan IIA reduced the population of Ki-67 positive cells, and the protein levels of p-Akt, c-Myc, and HK2 were decreased consistently (Fig. 6g). These results indicate that Tan IIA exhibits a potential anti-tumor effect in OSCC xenograft models.

To determine the toxicity of Tan IIA in vivo, we examined mouse bodyweight with vehicle and Tan IIA treatment. The results showed that Tan IIA did not reduce body weight significantly (Supplementary Fig. 3A, B). Consistently, the blood analysis revealed that Tan IIA did not cause a significant increase or decrease of the RBC and WBC counts. Furthermore, the functions of vital organs, such as bone marrow, kidney, and liver, were unaffected. The Hb, AST, ALT, and BUN were similar in vehicle- and Tan IIA-treated mice (Supplementary Fig. 3C). The maximum tolerated dose (MTD) of Tan IIA remains to be determined but the compound was tested at a dose that was safe and effective in mice.

## Discussion

Oral cancer is currently the sixth most common cancer and ranks eighth among the most common causes of cancer-related deaths worldwide^1,2^. Over 90% of oral cancers are diagnosed as OSCC, which are preferred to occur on the tongue. The mechanisms of OSCC oncogenesis are complicated, multiple risk factors, such as alcohol consumption and tobacco use, have been identified to relate to this malignancy^5,25^. Recent studies revealed that the deregulation of glucose metabolism plays a crucial role in the tumorigenesis of OSCC. The immunoregulatory protein B7-H3 promotes aerobic glycolysis in OSCC via PI3K/Akt/mTOR signaling^26^. Upregulation of glucose transporter-1 (Glut1) induces the metabolic shift from the tricarboxylic acid cycle to aerobic glycolysis^27^. Moreover, overexpression of PKM2 associated with the aggressive clinicopathological features and unfavorable prognosis in OSCC^28^. Activation of EGF^29^ and HGF^30^ signaling promote glycolytic reprogramming in OSCC. The promotion of glycolysis and the Warburg effect induces the cancer stem-like cell properties of human OSCC cells^29^. In addition, overexpression of HK2 enhances the metastatic potential of tongue squamous cell carcinoma via the SOD2-H_2_O_2_ pathway^31^. In the present study, we found that HK2 is overexpressed in OSCC tissues and cell lines, depletion of HK2 reduces the tumorigenic properties of OSCC cells in vitro and in vivo. Our data showed that suppression of HK2-mediated glycolysis with small compounds, Tan IIA, inhibits OSCC cell growth and tumor formation. These results indicate that the deregulation of glycolysis is a hallmark of human OSCC.

Tan IIA is one of the most important lipophilic components extracted from *Salvia miltiorrhiza Bunge*. Tan IIA exhibits significant pharmacological activities on multiple human diseases, such as inhibition of left ventricular hypertrophy and attenuation of atherosclerosis^32,33^. Recently, Tan IIA has gained increasing attention due to the potential anti-tumor efficacy in various cancer models. Tan IIA suppresses both hematological and solid tumors, including leukemia^34^, non-small-cell lung cancer^35^, colorectal cancer^36^, prostate cancer^37^, and hepatocellular carcinoma^38^. The mechanism study revealed that treatment with Tan IIA-induced cell cycle arrest and apoptosis suppressed angiogenesis and metastasis, and enhanced the anti-tumor effect of chemotherapy agents^33^. Importantly, Tan IIA inhibits glucose metabolism leading to apoptosis in cervical cancer^39^. However, the inhibitory effect of Tan IIA on OSCC remains unclear. We revealed that suppression of Akt-c-Myc-HK2 axis played a critical role in Tan IIA-induced anti-tumor activity. Currently, a panel of natural products, including garcinol^40^, Salvianolic acid B^41^, and xylitol^42^, have been identified to suppress OSCC cells via inhibition of glycolysis. Moreover, attenuation of HK2-mediated glycolysis by small compounds impaired the malignant phenotypes of various human cancers, such as non-small cell lung cancer and colon cancer^43–45^. These evidences suggest that targeting glycolysis is a promising anti-tumor strategy which deserves further study.

Overexpression of HK2 promotes cancer cell survival, in which tumor cells may escape apoptosis and closely related to chemo/radiotherapy resistance. For example, the E3 ligase Skp2 increases the mitochondrial localization of HK2 and drives tumor growth and chemoresistance to cisplatin^46^. HK2 confers cisplatin resistance in ovarian cancer cells by enhancing cisplatin-induced autophagy^47^. Furthermore, B7-H3 promotes aerobic glycolysis and chemoresistance in colorectal cancer cells in an HK2-dependent manner^48^. Knockdown of LncRNA-UCA1 suppresses chemoresistance of pediatric AML by inhibiting glycolysis through the microRNA-125a/hexokinase 2 pathway^49^. Also, depletion of HK2 increases the sensitivity to radiotherapy in glioma^50^, cervical cancer^51^, and laryngeal carcinoma^52^. Our data showed that the natural product Tan IIA inhibits HK2 expression in OSCC cells, and activates the mitochondrial apoptosis signaling. Overexpression of HK2 compromises Tan IIA-induced cell death and restores glycolysis in OSCC cells.

HK2 is regulated at the transcriptional level by the transcription factors c-Myc and HIF-1α^23,53^. Furthermore, the non-coding RNAs, such as long intergenic non-coding RNAs (lincRNA-RoR)^54^ and miRNA (miR-218)^55^ also required for efficient HK2 expression. Our data indicate that Akt activity plays a predominant role in promoting of HK2 expression in OSCC cells by regulation of c-Myc stability. Tan IIA inhibits Akt phosphorylation and promotes the interaction between c-Myc and E3 ligase FBW7, which eventually enhances FBW7-mediated c-Myc ubiquitination and degradation. Overexpression of Akt restored the expression of c-Myc and HK2 in OSCC cells and compromised Tan IIA-induced glycolysis suppression.

Overall, this study suggests that high expression of HK2 is required for maintaining the malignant phenotype of OSCC cells. The natural product Tan IIA inhibits OSCC cells by reducing of glycolysis in an Akt-c-Myc-HK2 signaling-dependent manner. This evidence extends our understanding of the anti-tumor mechanism of Tan IIA and indicates Tan IIA a potential anti-tumor agent for OSCC prevention and treatment.

## Supplementary information

Supplementary Figure legend

Supplementary Figure 1

supplementary figure 2

supplementary figure 3

supplementary table 1

supplementary table 2
