# Supplementary material for: Tanshinone IIA inhibits oral squamous cell carcinoma via reducing Akt-c-Myc signaling-mediated aerobic glycolysis
Source: Cell Death Dis. 2020 May 18;11(5):381. doi: 10.1038/s41419-020-2579-9 (PMC7235009; doi:10.1038/s41419-020-2579-9)
Supplement: Supplementary file 1 — Supplementary Figure legend [file 41419_2020_2579_MOESM1_ESM.docx]

**Supplementary Figure legends**

Figure 1. HK2 promotes glycolysis in OSCC cells. A and B, Glucose consumption (A) and lactate production (B) in CAL27 and SCC15 cells expressing Ctrl or HK2 sgRNA. C, Immunoblotting (IB) analysis of HK2 in hTERT-OME cells with control vector or HK2 transfection. D, Glucose consumption and lactate production of hTERT-OME cells with control vector or HK2 transfection. ****p*<0.001. E and F, Trypan blue exclusion assay analysis of the population of live cells in Tan IIA-treated OSCC cells with various time points (E) or concentrations (F). ****p*<0.001.

Figure 2. The effect of Tanshinone IIA (Tan IIA) on O_2_ consumption of OSCC cells. CAL27 and SCC15 cells were treated with Tan IIA and subjected to O_2_ consumption analysis. **p*<0.05, ***p*<0.01, ****p*<0.001.

Figure 3. Toxicity analysis of Tan IIA treated xenograft model. A and B, Body weights of CAL27 (A), and SCC15 (B) xenograft tumor-bearing mice with the vehicle and Tan IIA treatment. C, Blood analysis of tumor-bearing mice with vehicle or Tan IIA treatment.
