# Supplementary figures and images for: Tanshinone IIA inhibits oral squamous cell carcinoma via reducing Akt-c-Myc signaling-mediated aerobic glycolysis
Source: Cell Death Dis. 2020 May 18;11(5):381. doi: 10.1038/s41419-020-2579-9 (PMC7235009; doi:10.1038/s41419-020-2579-9)

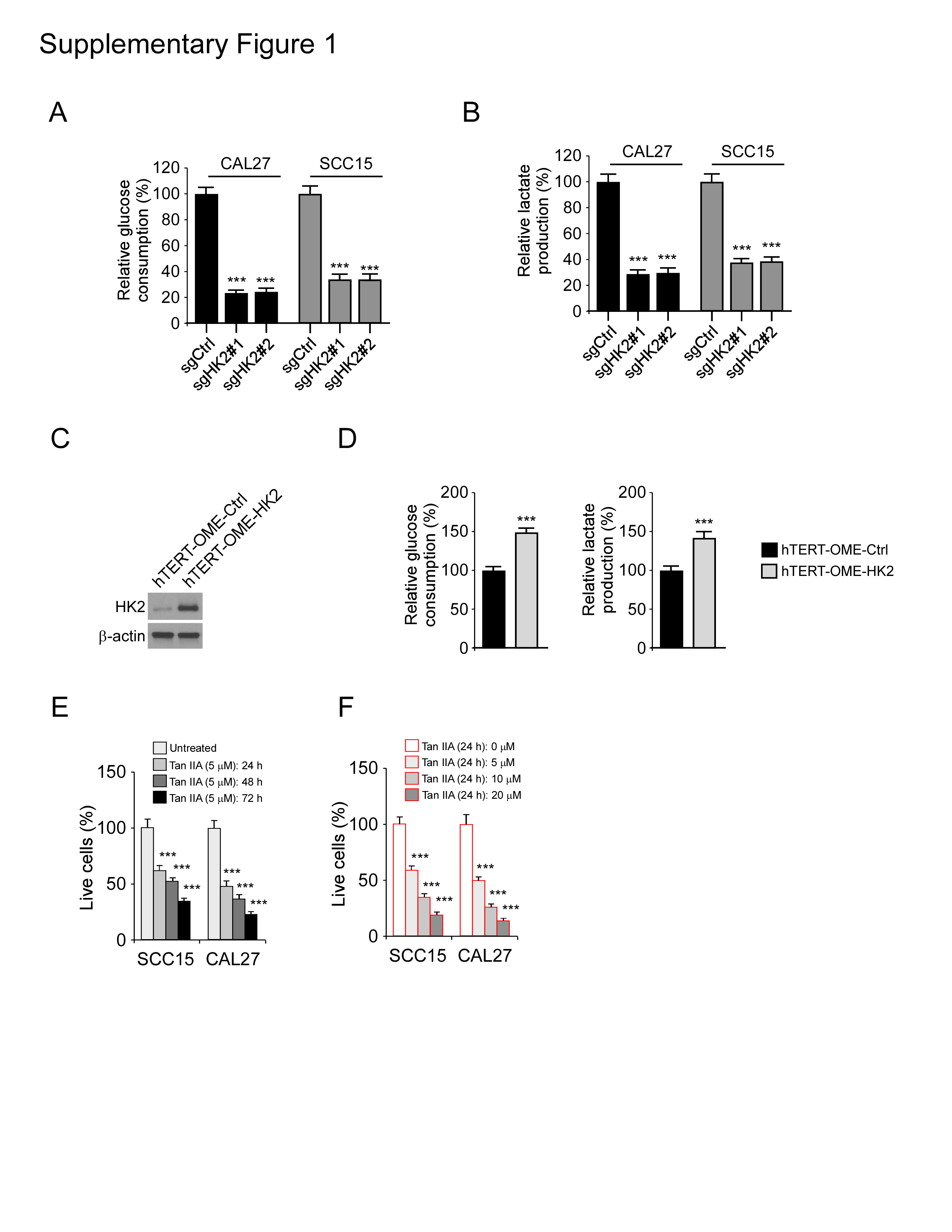

Supplement: Supplementary file 2 — Supplementary Figure 1 [file 41419_2020_2579_MOESM2_ESM.png]

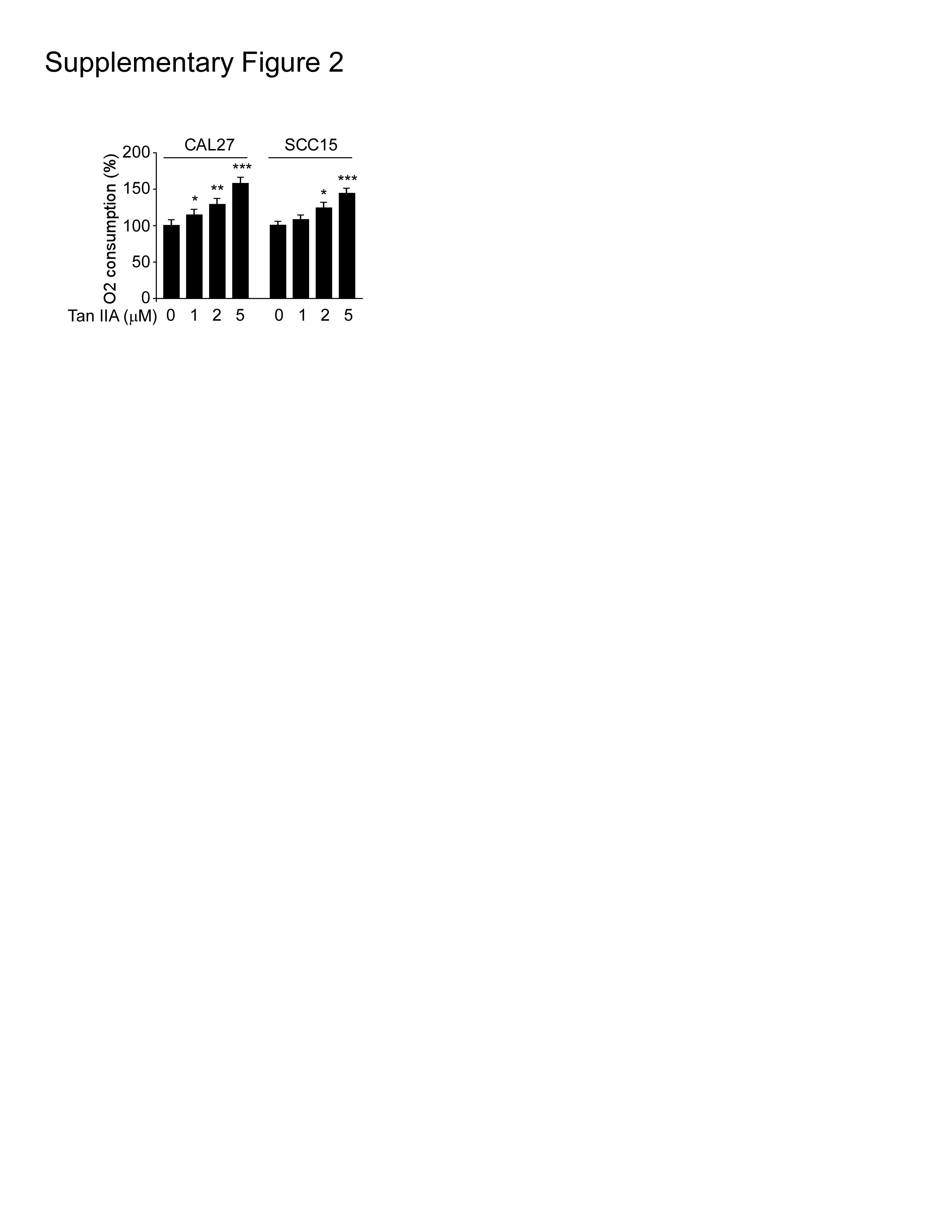

Supplement: Supplementary file 3 — supplementary figure 2 [file 41419_2020_2579_MOESM3_ESM.png]

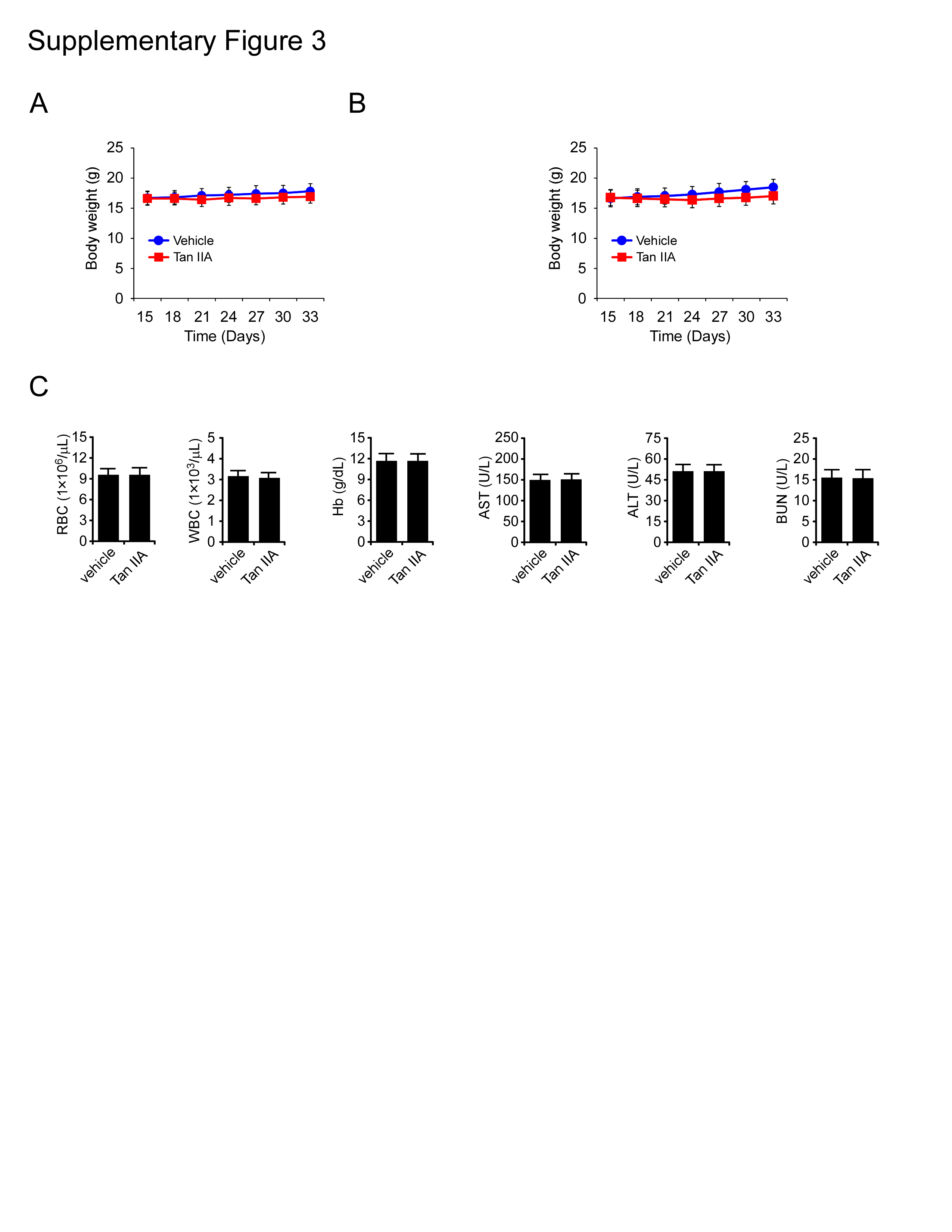

Supplement: Supplementary file 4 — supplementary figure 3 [file 41419_2020_2579_MOESM4_ESM.png]
