# Supplementary material for: Tanshinone IIA inhibits oral squamous cell carcinoma via reducing Akt-c-Myc signaling-mediated aerobic glycolysis
Source: Cell Death Dis. 2020 May 18;11(5):381. doi: 10.1038/s41419-020-2579-9 (PMC7235009; doi:10.1038/s41419-020-2579-9)
Supplement: Supplementary file 5 — supplementary table 1 [file 41419_2020_2579_MOESM5_ESM.docx]

**Supplementary Table 1. Screened compound list**

| **Compound** | **Glucose uptake** | | |  | **Lactate production** | | |
| --- | --- | --- | --- | --- | --- | --- | --- |
|  | **A** | **B** | **C** |  | **A** | **B** | **C** |
| DMSO | 100 | 100 | 100 |  | 100 | 100 | 100 |
| Apigenin | 101 | 106 | 104 |  | 106 | 101 | 105 |
| Cytisine | 101 | 103 | 101 |  | 98 | 98 | 94 |
| Icariin | 98 | 99 | 103 |  | 94 | 96 | 97 |
| Neohesperidin | 94 | 93 | 98 |  | 97 | 95 | 93 |
| Salicin | 92 | 91 | 92 |  | 87 | 93 | 89 |
| Tangeretin | 97 | 91 | 93 |  | 94 | 94 | 95 |
| (-)-Epigallocatechin Gallate | 82 | 88 | 86 |  | 86 | 84 | 84 |
| Arbutin | 95 | 93 | 95 |  | 91 | 92 | 87 |
| Bilobalide | 92 | 101 | 93 |  | 98 | 95 | 91 |
| Gossypol | 102 | 93 | 95 |  | 95 | 92 | 97 |
| Nobiletin | 96 | 95 | 99 |  | 92 | 94 | 92 |
| Phloretin | 100 | 96 | 93 |  | 95 | 94 | 87 |
| Sclareol | 84 | 83 | 86 |  | 84 | 89 | 87 |
| Tanshinone I | 94 | 97 | 97 |  | 91 | 92 | 94 |
| Artesunate | 97 | 101 | 103 |  | 94 | 97 | 96 |
| Caffeic Acid | 101 | 103 | 102 |  | 99 | 96 | 98 |
| Gramine | 93 | 94 | 92 |  | 89 | 89 | 94 |
| Kaempferol | 91 | 92 | 95 |  | 95 | 95 | 91 |
| Phlorizin | 102 | 98 | 101 |  | 94 | 99 | 96 |
| Sclareolide | 96 | 91 | 98 |  | 95 | 89 | 91 |
| Tanshinone IIA | 72 | 71 | 72 |  | 66 | 68 | 73 |
| Emodin | 98 | 93 | 91 |  | 93 | 85 | 86 |
| Kinetin | 103 | 102 | 103 |  | 106 | 102 | 101 |
| Myricetin | 93 | 92 | 94 |  | 91 | 87 | 86 |
| Oridonin | 102 | 94 | 96 |  | 97 | 83 | 94 |
| Piperine | 89 | 92 | 84 |  | 84 | 85 | 91 |
| Taxifolin (Dihydroquercetin) | 93 | 94 | 95 |  | 89 | 84 | 87 |
| Azomycin | 104 | 94 | 97 |  | 95 | 96 | 93 |
| Chrysin | 88 | 93 | 94 |  | 85 | 93 | 95 |
| Enoxolone | 102 | 107 | 97 |  | 106 | 101 | 103 |
| Hesperetin | 104 | 98 | 107 |  | 102 | 110 | 103 |
| Myricitrin | 98 | 89 | 96 |  | 93 | 93 | 94 |
| Puerarin | 86 | 89 | 94 |  | 93 | 91 | 93 |
| Silibinin | 94 | 92 | 91 |  | 91 | 83 | 93 |
| Esculin | 98 | 99 | 103 |  | 89 | 93 | 97 |
| Baicalein | 92 | 85 | 92 |  | 95 | 91 | 87 |
| **Compound** | **Glucose uptake** | | |  | **Lactate production** | | |
|  | **A** | **B** | **C** |  | **A** | **B** | **C** |
| Cinchonidine | 91 | 97 | 93 |  | 86 | 87 | 93 |
| Fisetin | 101 | 102 | 98 |  | 98 | 94 | 91 |
| Hesperidin | 94 | 92 | 96 |  | 85 | 91 | 94 |
| Limonin | 103 | 101 | 106 |  | 98 | 104 | 101 |
| Osthole | 94 | 91 | 89 |  | 91 | 89 | 87 |
| Quercetin Dihydrate | 93 | 91 | 96 |  | 93 | 94 | 92 |
| Silymarin | 91 | 93 | 93 |  | 94 | 91 | 91 |
| Troxerutin | 91 | 94 | 93 |  | 92 | 90 | 89 |
| Rutaecarpine | 95 | 96 | 98 |  | 94 | 97 | 89 |
| Oxymatrine | 111 | 106 | 108 |  | 102 | 104 | 101 |
| Sinomenine | 103 | 107 | 98 |  | 102 | 106 | 101 |
| Naringin | 103 | 101 | 97 |  | 94 | 98 | 102 |
| Luteolin | 101 | 103 | 98 |  | 94 | 92 | 102 |
| Honokiol | 93 | 91 | 88 |  | 94 | 89 | 92 |
| Formononetin | 89 | 89 | 94 |  | 93 | 92 | 86 |
| Cryptotanshinone | 94 | 98 | 101 |  | 92 | 95 | 97 |
| Baicalin | 103 | 103 | 98 |  | 102 | 105 | 102 |
| Laetrile | 104 | 106 | 101 |  | 102 | 102 | 105 |
| Andrographolide | 94 | 96 | 93 |  | 89 | 89 | 92 |
| Cyclosporin A | 94 | 92 | 91 |  | 92 | 87 | 85 |
| Bergenin | 114 | 112 | 112 |  | 102 | 106 | 107 |
| Magnolol | 98 | 99 | 104 |  | 95 | 102 | 97 |
| Paeonol | 94 | 95 | 97 |  | 89 | 86 | 93 |
| Rutin | 103 | 103 | 95 |  | 102 | 92 | 98 |
| Synephrine | 89 | 93 | 96 |  | 93 | 91 | 94 |
| Vanillylacetone | 91 | 81 | 87 |  | 89 | 92 | 94 |
| Xanthone | 87 | 86 | 84 |  | 87 | 84 | 91 |
| Gastrodin | 102 | 107 | 106 |  | 94 | 105 | 102 |
| Quercetin | 91 | 92 | 87 |  | 92 | 95 | 91 |
| Isoliquiritigenin | 82 | 84 | 87 |  | 83 | 81 | 94 |
| Rotundine | 93 | 98 | 98 |  | 83 | 89 | 93 |
| Piperlongumine | 82 | 76 | 77 |  | 82 | 80 | 71 |
| Hematoxylin | 94 | 91 | 96 |  | 93 | 92 | 84 |
| Sesamin | 112 | 108 | 105 |  | 101 | 104 | 106 |
| Sophocarpine | 102 | 98 | 103 |  | 104 | 102 | 105 |
| Guanosine | 93 | 83 | 91 |  | 97 | 95 | 91 |
| Hordenine | 92 | 93 | 94 |  | 89 | 83 | 99 |
| Sorbitol | 103 | 101 | 105 |  | 104 | 102 | 101 |
| Inosine | 102 | 104 | 92 |  | 98 | 95 | 98 |
| Licochalcone A | 87 | 86 | 85 |  | 91 | 88 | 87 |
| **Compound** | **Glucose uptake** | | |  | **Lactate production** | | |
|  | **A** | **B** | **C** |  | **A** | **B** | **C** |
| Aloin | 103 | 105 | 96 |  | 102 | 102 | 106 |
| Indirubin | 94 | 99 | 103 |  | 94 | 91 | 92 |
| Naringenin | 89 | 92 | 94 |  | 93 | 89 | 93 |
| Curcumol | 91 | 84 | 89 |  | 93 | 91 | 85 |
| Vanillin | 93 | 97 | 91 |  | 89 | 86 | 94 |
| Oleuropein | 102 | 93 | 95 |  | 93 | 96 | 94 |
| Salidroside | 94 | 91 | 85 |  | 96 | 92 | 95 |
| Apocynin | 96 | 93 | 86 |  | 87 | 83 | 87 |
| Diosmetin | 83 | 87 | 94 |  | 87 | 92 | 92 |
| Dihydromyricetin | 89 | 78 | 83 |  | 88 | 81 | 86 |
| Triptolide | 94 | 96 | 94 |  | 89 | 87 | 93 |
| Formononetin | 84 | 87 | 81 |  | 86 | 82 | 84 |

A, replicate A; B, replicate B; C, replicate C;
