# Supplementary material for: Tanshinone IIA inhibits oral squamous cell carcinoma via reducing Akt-c-Myc signaling-mediated aerobic glycolysis
Source: Cell Death Dis. 2020 May 18;11(5):381. doi: 10.1038/s41419-020-2579-9 (PMC7235009; doi:10.1038/s41419-020-2579-9)
Supplement: Supplementary file 6 — supplementary table 2 [file 41419_2020_2579_MOESM6_ESM.docx]

**Supplementary Table 2. sgRNAs sequence**

| **Name** | **Forward** | **Reverse** |
| --- | --- | --- |
| **sgHK2 #1** | GATGACCACAACTGTGAGAT | ATCTCACAGTTGTGGTCATC |
| **sgHK2 #2** | ATCATAACCACAGGTCATCA | TGATGACCTGTGGTTATGAT |
| **sgc-Myc #1** | AACGTTGAGGGGCATCGTCG | CGACGATGCCCCTCAACGTT |
| **sgc-Myc #2** | TGCGTAGTTGTGCTGATGTG | CACATCAGCACAACTACGCA |
| **sgAkt1 #1** | TCACGTTGGTCCACATCCTG | CAGGATTGGACCAACGTGA |
| **sgAkt1 #2** | GCAGGATGTGGACCAACGTG | CACGTTGGTCCACATCCTGC |
|  |  |  |
